# Effect of oscillating feeding time and corn processing on performance and carcass characteristics of feedlot steers

**DOI:** 10.1093/tas/txaa103

**Published:** 2020-06-26

**Authors:** Alejandro E Relling, David Douglas Clevenger, Francis L Fluharty

**Affiliations:** 1 Department of Animal Sciences, The Ohio State University, Wooster, OH; 2 Department of Animal and Dairy Science, University of Georgia, Athens, GA

**Keywords:** alternate feeding time, bunk management, corn processing, mixing variability

## Abstract

The objectives of this experiment were to evaluate the effect of oscillating feeding time (OFT) and corn processing (CoP) on performance and carcass characteristics of feedlot cattle. One hundred sixty-five steers (initial body weight [BW] 277.2 ± 27.80 kg) were blocked by initial BW and allotted to 24 pens. Pens within each block were randomly assigned based on a 2 × 2 factorial arrangement of treatments. The two factors were CoP (whole shelled corn vs. ground corn [GC]) and feeding time (FT) (constant FT vs. 2 hours OFT). Animals were fed the same diet, only changing the CoP method depending on the treatment. Feed offered and feed refusals were collected daily. Body weight was collected at starting day of the experiment (d1) and every 28 d until the end of the experiment. At the end of the experiment, animals were harvested in a commercial slaughter facility, and carcass data were collected by a USDA grader. Animal growth performance and carcass characteristics data were analyzed with the PROC Mixed procedure (SAS) using CoP, FT, and their interaction as fixed variables; and treatments × pen within each block and block were considered random variables. There was no CoP by FT interaction differences (*P* > 0.10) on animal growth performance variables, nor on hot carcass weight, back fat, rib eye area, or the percentage of kidney, pelvic, and heart fat. There was a tendency for an interaction (*P* = 0.08) for marbling score, where the steers from the GC processing fed on the oscillating time had a lesser marbling score than the other three treatments. Oscillating feeding time improved (*P* = 0.05) average daily gain; but did not affect (*P* ≥ 0.11) dry matter intake (DMI), nor carcass characteristics compared with feeding at a similar time every day. There were no effects (*P* ≥ 0.11) of CoP on growth performance, nor carcass characteristics. In conclusion, a 2-hour oscillation in FT might not decrease steer feedlot performance. This may be due to the high degree of control of DMI with feed bunk management.

## INTRODUCTION

Nutritional management is the most efficient way to avoid acidosis in feedlot cattle (Pritchard and Bruns, 2003). One of the most common management practices is bunk management, which is the controlled approach of feed delivery to cattle (Pritchard and Bruns, 2003). Bunk management includes the delivery of feed in a very constant way considering quantity, quality, and time (Galyean et al., 1992; Pritchard and Bruns, 2003). The concept of feeding management is something that has been around for over a century (Mumford, 1907); and it has been considered that 15 min changes in feed delivery may cause decreases in performance, and that the magnitude of the decrease depends on the extent and frequency of the irregularity (Mumford, 1907). In a previous experiment (Relling et al., 2017), we found that an oscillation of 2 hours per day on the feeding time (FT) does not affect growth (overall body weight [BW] or average daily gain [ADG]), dry matter intake (DMI), feed efficiency (ADG/DMI ratio [G:F]), or carcass characteristics in feedlot cattle. The lack of differences on performance observed previously (Relling et al., 2017) could be due to the type of processing of the corn. In that previous experiment (Relling et al., 2017), whole shelled corn (WSC) was used. Cracked or ground corn (CC or GC, respectively) is more completely fermented in the rumen compared with WSC (Owens et al., 1997); therefore, the used of WSC instead of GC might decrease the risk of acidosis in the experiment presented by Relling et al. (2017). Despite the importance of bunk management, FT, and corn processing (CoP) in feedlot performance, there are no experiments evaluating the effect of oscillating feeding time (OFT) with different CoP methods.

The hypothesis of the current experiment was that WSC prevented the decrease in growth performance when feedlot steers are fed at an oscillating time compared with steers fed GC at an oscillating time. The objective of the current experiment was to evaluate the effect of oscillating time of feeding and CoP on growth performance and carcass characteristics in feedlot cattle.

## MATERIALS AND METHODS

All procedures involving animals were approved by The Ohio State University Agricultural Animal Care and Use Committee (IACUC # 2015A00000113).

### Animals, Experimental Design, and Treatments

The feedlot trial was conducted at The Ohio State University feedlot in Wooster, OH. One hundred sixty-eight Angus crossbred steers were started in an adaptation diet, from which 165 steers (initial BW 277.2 ± 27.80 kg; 8.3 ± 0.5 mo of age) were used in the project. Steers were blocked by initial BW into 2 BW blocks and allotted to 24 pens (12 pens per block). Twenty-one of the pens had 7 heads per pen, with the exception of 2 pens on the light block and 1 pen of the heavy block. Pens within each block were then randomly assigned to 1 of 4 treatments within a 2 × 2 factorial arrangement of treatments (3 pens per treatment per block). The two main factors were: 1) FT and 2) CoP. The two treatments for FT were the control (CONT), animals in this group received the diet at the same time of the day (starting at 0900 the lighter steers’ block and at 1200 the heavier steers’ block), and OFT, these animals received the same diet as the control diet, but they were fed one hour earlier on the odd days of the experiment and 1 hour later on the even day of the experiment (0800 and 1000 for the lighter steers’ block and 1100 and 1300 for the heavier steers’ block). The two treatments for CoP were WSC and GC. The same lot of corn was used for the WSC and GC diets. The feed ingredients and nutrient composition of the diets are presented in Table 1. All diets were designed to meet or exceed dietary requirements (NRC, 2000) for growing and finishing beef steers. Feed was offered using a clean bunk management in which feed offered was increased 5% (DM basis), if bunks were clean 2 d in a row. For the CONT, the bunk reading was done daily; and for the OFT, the reading and change of feed offered was done on the even days if it was needed. The amount fed the odd days was the same as the previous day. The change of diet from growing to finishing was accomplished in 3 wk by removing 10% of the corn silage and adding 10% of corn (WSC or GC, depending on the corn CoP treatment) per week. The amount of feed offered on the days of the diet switch was decreased by 5% (DM basis) compared with the previous day DMI and remained fixed for 2 consecutive days before the 5% increase was reapplied.

**Table 1.** Dietary and nutrient composition of growing and finishing phase diets (% DM basis)

	Growing diet		Finishing diet	
Item	WSC^1^	GC^1^	WSC^1^	GC^1^
Whole corn	20	—	50	—
Ground corn	4.11	24.11	6.460	56.460
Corn silage	50	50	20	20
DDGS^2^	20	20	20	20
Soybean meal	2.0	2.0	0	0
Urea	0.5	0.5	0.369	0.369
Limestone	1.800	1.800	1.661	1.661
Rumensin 90^3^	0.017	0.017	0.020	0.020
Trace mineral salt^4^	1.536	1.536	1.455	1.455
Vitamin A, 30,000 IU/g	0.0074	0.0074	0.007	0.007
Vitamin D, 3,000 IU/g	0.0074	0.0074	0.007	0.007
Vitamin E, 44 IU/g	0.0222	0.0222	0.021	0.021
Nutrient composition				
CP, %	15.67	15.53	14.52	14.18
NDF, %	26.02	26.27	16.33	16.95
ADF, %	13.18	13.23	8.35	8.48
EE, %	3.60	3.41	4.15	3.67
Ash, %	6.06	6.00	5.64	5.48

EE, ether-extractable.

^1^GC, ground corn; WSC, whole shelled corn.

^2^Dakota Gold, Marion OH.

^3^Rumensin 90 (Elanco Animal Health, Greenfield, IN) provided 30 or 40 mg of monensin per kg/feed during the growing and finishing phases, respectively.

^4^Trace mineral salt contain 31.78% of sodium chloride, 44.48% of calcium sulfate, 19.06% potassium chloride, 0.38% of copper sulfate, 1.27% of zinc sulfate, 0.61% of magnesium sulfate, 0.007% of cobalt carbonate and 2.41% of Se for the growing phase, and 31.74% of sodium chloride, 44.40% of calcium sulfate, 19.03% potassium chloride, 0.41% of copper sulfate, 1.27% of zinc sulfate, 0.76% of magnesium sulfate, 0.007% of cobalt carbonate, and 2.41% of selenium for the finishing phase.

### Sampling and Analysis

Feed was offered daily and feed refusals were weighed, recorded, and discarded daily for the CONT, and every other day for the OFT, if some were in the bunk. Fresh water was available at all time. Feed samples were analyzed weekly for DM to allow determination of DMI. Weekly composite feed samples were dried in a forced-air oven at 55 °C and stored for future analysis.

Steers were individually weighed at day 0 for blocking and at the start of the experiment (d1), then every 28-d during the trial until d168 of the trial. Initial BW was considered the average of d0 and d1 BW. The finishing weight was taken on d188 and d203 for the heavier and lighter BW steers’ blocks, respectively. The animals in each pen and block were closed out together were on 1 d: making a total of 2 close out days, d188 for the heavier block and d203 for the lighter block. Steers were weighed before feeding and were not withheld from feed or water.

When steers had approximately 1.2 cm of back fat (visual appraisal; Phillips et al., 2002; Felix and Loerch, 2011), they were weighed, and harvested at a commercial abattoir. Hot carcass weight (HCW), *Longissimus dorsi* muscle area (LMA), marbling score, kidney, pelvic, and heart fat (KPH), and United State Department of Agriculture (USDA) quality grade (QG; USDA, 1997) were determined by a USDA inspector. Hot carcass weight was recorded on the day of slaughter and dressing percentage was calculated. Carcasses were chilled for 48 hours at −4 °C and ribbed between the 12th and 13th ribs to determine subcutaneous back fat thickness at the 12th rib (BF), LMA, marbling scores, KPH, and USDA QG.

Composited feed samples were analyzed for DM (100 °C for 24 hours), acid detergent fiber (ADF) and neutral detergent fiber (NDF) (Ankom Technology method 5 and 6, respectively; Ankom Technology, Fairport, NY), crude protein (CP) (method 930.15; AOAC, 1996), ether-extractable lipid (method 2; Ankom Technology, Fairport, NY), and total ash (600 °C for 12 hours).

### Statistical Analysis

Growth performance and carcass data were analyzed as a randomized complete block design with a 2 × 2 factorial arrangement of treatments using the MIXED procedure of SAS, version 9.4 (SAS Institute, Inc., Cary, NC, 2005). The blocking criteria were initial BW. The model included the two main factors (FT and CoP) treatments and interactions between them as fixed variables and treatments (FT × CoP) × pen within each block (experimental unit) and block as random variables. Because different final days on feed were used in each block, the actual days of the experiment that the animals were weighed was added as a covariable for the carcass data. Data were presented in a table format showing the LSMeans and a pooled SEM. The effects of fixed factors were declared significant at *P* ≤ 0.05, and tendencies are discussed at 0.05 < *P* ≤ 0.10. If the interaction was significant, the PDIFF option of SAS was used for mean separation when the *P*-value for an interaction was ≤0.10.

## RESULTS AND DISCUSSIONS

During the growing phase and finishing phases, there were no CoP by FT differences on BW, ADG, DMI, or G:F (*P* ≥ 0.19; Table 2); therefore, the growth performance results will be presented as the main effect of FT or CoP (Table 2). Corn processing did not affect (*P* ≥ 0.38; Table 2) BW, ADG, DMI, nor G:F. The growth performance results from the current experiment differ from previous results (Corona et al., 2005; Gorocica-Buenfil and Loerch, 2005; Freitas et al., 2020). Corona et al. (2005) reported an increase in DMI in the steers fed GC compared with the ones fed whole corn. A difference between the current and Corona et al. (2005) experiment is that in the later experiment, the animals were implanted and the steers in the current experiment were not. Gorocica-Buenfil and Loerch (2005) compared CoP and fiber length; and they found the main effect of CoP on ADG and G:F associated with a greater ADG and feed efficiency for the whole corn fed group compared with the CC fed group at the end of the finishing period. Similar results have been reviewed previously (Owens et al., 1997). As for the experiment by Corona et al. (2005), the steers used by Gorocica-Buenfil and Loerch (2005) were implanted. Freitas et al. (2020) observed a reduction of DMI and an improvement in G:F in steers fed CC compared with WSC. Cattle in the Freitas et al. (2020) experiment were not implanted animals, as in the current experiment. However, the animals used by Freitas et al. (2020) were backgrounded, and the ones in the current experiment were not. Therefore, the effect of CoP in growth performance might be multifactorial, and factorial arrangement experiment designs need to be conducted to evaluate the interaction of these factors, such as age, previous diet, and the use of implants.

**Table 2.** Effect of different FT and CoP (during the growing [D56] or finishing [Finishing] phases on BW, ADG, DMI, and gain to feed ratio [G:F] in feedlot steers)

	Feeding time		Corn processing			*P*-values^2^		
Treatments	CONT^1^	OFT^1^	GC^1^	WSC^1^	SEM	FT	CoP	FT × CoP
Pens^3^	12	12	12	12				
Body weight, kg								
Initial	277.1	277.3	277.1	277.2	24.69	0.74	0.81	0.40
D56 BW	371.8	372.4	370.9	373.2	25.16	0.80	0.38	0.56
Finishing BW	562.3	569.9	565.4	566.8	12.69	0.23	0.82	0.62
ADG, kg/d								
D56 ADG	1.69	1.70	1.67	1.71	0.031	0.76	0.38	0.46
Finishing ADG	1.41	1.50	1.45	1.46	0.046	0.05	0.77	0.40
DMI, kg/d								
D56 DMI	8.01	8.00	7.99	8.02	0.449	0.81	0.65	0.99
Finishing DMI	9.04	9.20	9.06	9.18	0.210	0.27	0.43	0.19
G:F								
D56 G:F	0.211	0.213	0.210	0.214	0.0118	0.62	0.45	0.44
Finishing G:F	0.156	0.164	0.160	0.160	0.0082	0.09	0.92	0.91

^1^CONT, constant feeding time; GC, ground corn; OFT, oscillating feeding time; WSC, whole shelled corn.

^2^ *P*-values for the main effects of FT, CoP, and the interaction of the main factors (CoP × FT).

^3^Twenty-one pens had 7 heads per pen, and 3 pens had 6 heads per pen. Those 3 pens were: one pen on CONT-GC and one pen for OFT-WSC, both on the light block, and one pen on CONT-GC on the heavy block.

We have shown (Relling et al., 2017) that a daily oscillation of 2 hours on FT did not impact feedlot cattle growth. In the current experiment, OFT did not affect growing or final BW, growing ADG, growing or finishing phase DMI, nor growing G:F. However, OFT in a 2-hour window improved (*P* = 0.05; Table 2) finishing ADG during the finishing phase, which trend to improve (*P* = 0.09) G:F during the finishing phase. This result does not support our hypothesis, and there is little literature that supports this finding. It is possible that using feed bunk management (Galyean et al., 1992; Pritchard and Bruns, 2003) in a robust feeding management scheme in which daily constant, and continuous, variations on the time of feeding (in 2-hour window) do not affect performance. There is little data on feeding behavior and animal growth. Inconsistent feeding behavior has been associated with decreases in growth rate, and an increased risk of ruminal acidosis (Schwartzkopf-Genswein et al., 2003). Also, calmer cattle grow faster than stressed/excitable cattle (Olson et al., 2019). Changing FT might be a factor that stresses the animals; however, providing enough feed without drastic changes in DMI might ameliorate the effect of changing FT in a programmed and systematic manner. Previously, Cooper et al. (1998) reported that a 4-hour delay in FT as a single event decreased rumen pH the following 6 d up to a point of subacute acidosis. However, this was a single event, during the finishing phase, and with a greater delay than what was evaluated in the current experiment. It is possible that the adaptation of the oscillating 2-hour difference in FT on a low-grain diet may reduce the digestive disorders that a change in FT, on nonadapted cattle on high-grain diets, may have. It is also worth mentioning that the recommendations on FT changes and the effects on animal growth (Mumford, 1907) occurred long before the establishment of the recommendations of feed bunk management (Galyean et al., 1992; Pritchard and Bruns, 2003); and no evaluations have been conducted since to evaluate the effect of oscillation of FT.

There were no FT, CoP, nor interaction effects for HCW, BF, LMA, KPH, yield grade (YG), nor QG (*P* ≥ 0.16; Table 3). The use of CC increased REA (Freitas et al., 2020), improved marbling score and the percentage of animals that were graded Prime (Gorocica-Buenfil and Loerch, 2005), and increased dressing percentage (Corona et al., 2005). The difference in carcass characteristics in the current experiment and those mentioned previously could be due to the age of the animals (backgrounded vs. not), the use of implants (implanted vs. not implanted), or variables that were not evaluated in any of the experiments, such as propionate absorption, glucose absorption, endocrine profile, and their interactions. All these variables might have an impact in muscle and adipose tissue accretion; and they could be affected by CoP (Owen et al., 1997). Feeding time did not affect carcass characteristics. This result confirms our previous finding (Relling et al., 2017), but it contradicts our hypothesis, based on the interaction of FT and CoP. As described previously on the growth performance, it is possible that the feed bunk management is a robust management practice that allowed a constant 2-hour window in the FT without affecting carcass characteristics. However, more studies are needed to test the effect of the OFT in rumen pH and feeding behavior among other measurements to confirm this finding.

**Table 3.** Effect of different FT and CoP on carcass characteristics of feedlot steers

CoP^1^	WSC^1^		GC^1^			*P*-values^2^		
FT^1^	CONT^1^	OFT^1^	CONT^1^	OFT^1^	SEM	FT	CoP	CoP × FT
Pens^3^	6	6	6	6				
HCW, kg	339.9	343.7	341.5	346.0	9.02	0.21	0.57	0.92
BF, cm	1.52	1.56	1.46	1.50	0.065	0.54	0.33	0.97
LMA, cm^2^	78.97	79.95	80.38	81.63	1.023	0.28	0.13	0.89
KPH, %	2.20	2.10	2.13	2.14	0.04	0.35	0.75	0.15
Marbling^4^	712.8^a^	715.5^a^	734.3^a^	675.8^b^	17.0	0.10	0.59	0.08
YG^5^	2.95	2.93	2.80	2.79	0.110	0.85	0.17	0.93

^1^CONT, constant feeding time; CoP, main effect of corn processing; FT, main effect of feeding time; GC, ground corn; OFT, oscillating feeding time; WSC, whole shelled corn.

^2^ *P*-values for the main effects of FT, CoP, and the interaction of the main factors (CoP × FT).

^3^Twenty-one pens had 7 heads per pen and 3 pens had 6 heads per pen. Those 3 pens were: one pen on CONT-GC and one pen for OFT-WSC, both on the light block, and one pen on CONT-GC on the heavy block.

^4^Marbling score scale: marbling 400–490 = slight, 500–590 = small, 600–690 = modest, 700–790 = moderate, 800–890 = slightly abundant.

^5^YG was calculated using the YG equation from the USDA beef grading standards (USDA, 1997).

^ab^Within a row, means without a common superscript letter differ, *P*-value <0.05. PDIFF option of SAS was used for mean separation.

Marbling score tended to decrease (CoP × FT interaction; *P* = 0.08; Table 3) due to a decrease in intramuscular fat deposition in the GC-OFT cattle compared with the cattle in the other three treatments. This change in marbling is not associated with any of the growth performance results of the present experiment. In the current experiment, we did not evaluate rumen pH or plasma biomarkers of animal metabolism; however, we may consider that GC-OFT arrangement of treatments produces a greater reduction in rumen pH than the other treatments, which may affect marbling score. This assumption is based in the studies that show the association between rumen pH and marbling score when corn distiller grains are fed to fattening feedlot cattle (Reinhardt et al., 2007; Felix et al., 2012). However, the lack of rumen pH measurements in the current experiment does not allow us to confirm it, nor to evaluate the lack of association between a decrease in marbling score, due to a possible drop in rumen pH, and growth performance.

In conclusion, the OFT within a 2-hour window does not affect growth, independently of the CoP; however, an oscillating feeding system tends to decrease marbling score when corn is processed, but not when it is fed as WSC.
